# Modeling heat transport in crystals and glasses from a unified lattice-dynamical approach

**DOI:** 10.1038/s41467-019-11572-4

**Published:** 2019-08-26

**Authors:** Leyla Isaeva, Giuseppe Barbalinardo, Davide Donadio, Stefano Baroni

**Affiliations:** 1 SISSA – Scuola Internazionale Superiore di Studi Avanzati, Trieste, 34136 Italy; 2 Department of Chemistry, University of California at Davis, Davis, CA 95616 USA; 3 CNR-IOM DEMOCRITOS, SISSA, Trieste, 34136 Italy

**Keywords:** Electronic properties and materials, Computational methods

## Abstract

We introduce a novel approach to model heat transport in solids, based on the Green-Kubo theory of linear response. It naturally bridges the Boltzmann kinetic approach in crystals and the Allen-Feldman model in glasses, leveraging interatomic force constants and normal-mode linewidths computed at mechanical equilibrium. At variance with molecular dynamics, our approach naturally and easily accounts for quantum mechanical effects in energy transport. Our methodology is carefully validated against results for crystalline and amorphous silicon from equilibrium molecular dynamics and, in the former case, from the Boltzmann transport equation.

## Abstract (web summary)

Heat transport is a key process in designing functional materials, yet its calculations remain challenging due to large variance in material structures. Isaeva et al. introduce a unified approach counting the quantum effects, which is capable of modeling heat transport ranging from crystals to glasses.

## Introduction

Heat transport in solid insulators, either crystalline or disordered, is dominated by the dynamics of lattice vibrations. Far from melting, atomic displacements from equilibrium are much smaller than interatomic distances and they can thus be treated in the (quasi-) harmonic approximation. In crystals, this observation enables a kinetic description of heat transport in terms of phonons, that can be embodied in the Peierls–Boltzmann transport equation (BTE)^1,2^. In disordered systems the typical phonon mean free paths may be so short that the quasi-particle picture of heat carriers breaks down and BTE is no longer applicable, making it necessary to resort to molecular dynamics (MD), in either its nonequilibrium or equilibrium (EMD) flavors^2,3^. MD is of general applicability to solids, either periodic or disordered, and liquids. Yet, it may require long simulation times and it is subject to statistical errors, which are at times cumbersome to evaluate especially for systems at low temperatures, where lack of ergodicity may be an issue. Most importantly, MD cannot account for quantum-mechanical effects^4^, which are instead easily treated in BTE, thus making the treatment of heat transport for glasses in the quantum regime, i.e. below the Debye temperature, a methodological challenge.

In this paper, we present a novel approach to heat transport in insulating solids, which combines the Green–Kubo (GK) theory of linear response^3,5–8^ and a quasi-harmonic description of lattice vibrations, thus resulting in a compact expression for the thermal conductivity, that unifies the BTE approach in the single-mode relaxation-time approximation (RTA) for crystals^2^ and a generalization of the Allen-Feldman (AF) model for disordered system^9,10^ that explicitly and naturally accounts for normal-mode lifetimes. The main ingredients of our approach are the matrix of the inter-atomic force constants (IFC) computed at mechanical equilibrium and the anharmonic lifetimes of the vibrational modes, as computed from the cubic corrections to the harmonic IFCs using the Fermi’s golden rule^11^. Our theory is thoroughly validated in crystalline and amorphous silicon by comparing its predictions with those of EMD simulations and BTE computations.

## Results

### Theory

The basis of our work is the GK theory of linear response^3,5–8^, which relates the heat conductivity to the ensemble average of the heat-flux auto-correlation function:

1 $$\kappa _{\alpha \beta } = \frac{1}{{Vk_{\mathrm{B}}T^2}}\mathop {\int}\limits_0^{ \infty } {\langle J_\alpha (t)J_\beta (0)\rangle } {\mathrm{d}}t,$$

where *V* and *T* are the system volume and temperature, *k*_B_ is the Boltzmann constant, *J*_*α*_(*t*) the *α*-th Cartesian component of the macroscopic heat flux, which in solids coincides with the energy flux, and 〈⋅〉 indicates a canonical average over initial conditions^3^. Far from melting, the energy flux and the lattice Hamiltonian of a solid, both crystalline and amorphous, can be expressed as power series in the atomic displacements, and Eq. (1) can be evaluated in terms of Gaussian integrals using standard field-theoretical techniques.

The energy flux can be expressed in terms of atomic positions, **R**_*i*_, and local energies, $\epsilon _i$, as ${\mathbf{J}} = \mathop {\sum}\limits_i {(\mathop {{\mathbf{R}}}\limits^. _i\epsilon _i + {\mathbf{R}}_i\dot \epsilon _i)}$ (ref. ^3^), where in the harmonic approximation $\epsilon _i$ can be defined as: $\epsilon _i = \frac{{M_i}}{2}\mathop {\sum}\limits_\alpha {\left( {\dot u_{i\alpha }} \right)^2} + \frac{1}{2}\mathop {\sum}\limits_{j\alpha \beta } {u_{i\alpha }} \Phi _{i\alpha }^{j\beta }u_{j\beta }$, *M*_*i*_ being the mass of *i*-th atom, ${\mathbf{u}}_i = {\mathbf{R}}_i - {\mathbf{R}}_i^ \circ$ its displacement from its equilibrium position, ${\mathbf{R}}_i^ \circ$, *α* and *β* label Cartesian components, and $\Phi _{i\alpha }^{j\beta } = \left. {\frac{{\partial ^2E}}{{\partial u_{i\alpha }\partial u_{j\beta }}}} \right|_{{\mathbf{u}} = 0}$ is the IFC matrix. By expressing the energy flux in terms of the **u**’s, one obtains: ${\mathbf{J}} = {\mathbf{J}}^ \circ + \frac{\mathrm{d}}{{\mathrm{d}}t}\mathop {\sum}\limits_i {{\mathbf{u}}_i} \epsilon _i$, where ${\mathbf{J}}^ \circ = \mathop {\sum}\limits_i {{\mathbf{R}}_i^ \circ } \dot \epsilon _i$. The second term on the right-hand side of this expression is the total time derivative of a process that, in the absence of atomic diffusion, is stationary and of finite variance. A recently established gauge invariance principle for heat transport^12,13^ states that such a total time derivative does not contribute to the thermal conductivity. We will therefore dispose of it and express the energy flux as: **J** ← **J**°. Note that it is essential to use equilibrium atomic positions in the definition of **J**°, i.e. the positions describing the (metastable) mechanical equilibrium of any given model of an ordered or disordered system, rather than instantaneous ones. Otherwise, the difference **J** − **J**° would not be a total time derivative of a stationary process and the value of the transport coefficient resulting from **J**° would be biased. By making use of Newton’s equation of motion, the final expression for the harmonic heat flux reads^9,10^:

2 $$J_\alpha = \frac{1}{2}\mathop {\sum}\limits_{ij\beta \gamma } {(R_{i\alpha }^ \circ - R_{j\alpha }^ \circ )} \Phi _{i\beta }^{j\gamma }u_{i\beta }\dot u_{j\gamma },$$

where the minimum-image convention is adopted for computing inter-atomic distances in periodic boundary conditions.

By inserting Eq. (2) into Eq. (1), the integrand is cast into the canonical average of a quartic polynomial in the atomic positions and velocities. In the harmonic approximation, this average reduces to a Gaussian integral, which can be evaluated by way of the Wick’s theorem^14^. By doing so, the resulting time integral would diverge, thus yielding an infinite conductivity, as expected for a harmonic crystal^15^. In order to regularize this integral, we introduce anharmonic effects by renormalizing the single-mode correlation functions using the normal-mode lifetimes, as explained below. Our final result for the heat conductivity tensor reads:

3 $$\kappa _{\alpha \beta } = \frac{{k_B}}{V}\mathop {\sum}\limits_{nm} {v_{nm}^\alpha } v_{nm}^\beta \tau _{nm}^ \circ ,$$

4 $$v_{nm}^\alpha = \frac{1}{{2\sqrt {\omega _n\omega _m} }}\mathop {\sum}\limits_{ij\beta \gamma } {\frac{{R_{i\alpha }^ \circ - R_{j\alpha }^ \circ }}{{\sqrt {M_iM_j} }}} \Phi _{i\beta }^{j\gamma }e_n^{i\beta }e_m^{j\gamma },$$

5 $$\tau _{nm}^ \circ = \frac{{\gamma _n + \gamma _m}}{{(\gamma _n + \gamma _m)^2 + (\omega _n - \omega _m)^2}} + {\cal{O}}(\epsilon ^2),$$

where *ω*_*n*_ and *γ*_*n*_ are the harmonic frequency and decay rate of the *n*-th normal mode, and $e_{ni}^\alpha$ is the corresponding eigenvector of the dynamical matrix, $\mathop {\sum}\limits_{j\beta } {\frac{1}{{\sqrt {M_iM_j} }}} \Phi _{j\beta }^{i\alpha }e_{nj}^\beta = \omega _n^2e_{ni}^\alpha$, and $\epsilon$ indicates the ratio *γ*/*ω*, which vanishes in the harmonic limit. Equations (3–5) will be dubbed as the quasi-harmonic Green-Kubo (QHGK) approximation for the heat conductivity.

In order to establish Eq. (3), we first express the energy flux in Eq. (2) in terms of normal-mode coordinates and momenta, defined as: $\xi _n = \mathop {\sum}\limits_{i\alpha } {\sqrt {M_i} } u_\alpha ^ie_{ni}^\alpha$ and $\pi _n = \mathop {\sum}\limits_{i\alpha } {\dot u_i^\alpha } e_{ni}^\alpha /\sqrt {M_i}$, reading: $J^\alpha = \mathop {\sum}\limits_{nm} {v_{nm}^\alpha } \sqrt {\omega _n\omega _m} \xi _n\pi _m$. It is then convenient to express these normal-mode coordinates and momenta in terms of classical complex amplitudes, reminiscent of the quantum boson ladder operators and defined as: $\alpha _n = \sqrt {\frac{{\omega _n}}{2}} \xi _n + \frac{i}{{\sqrt {2\omega _n} }}\pi _n$, whose time evolution is $\alpha _n(t) = \alpha _n(0){\mathrm{e}}^{ - i\omega _nt}$. By doing so, the energy flux can be expressed in terms of the *α* amplitudes as

6 $$J^\beta = \frac{i}{2}\mathop {\sum}\limits_{nm} {v_{nm}^\beta } \omega _m(\alpha _n^ \ast + \alpha _n)(\alpha _m^ \ast - \alpha _m).$$

By using this expression, the integrand in Eq. (1) becomes a Gaussian integral of a fourth-order polynomial in the *α*’s and α*’s that, by means of the Wick’s theorem^14^, can be cast into a sum of products of pairs of single-mode (classical) Green’s functions, $\langle \alpha _n^ \ast (t)\alpha _m(0)\rangle = \delta _{nm}g_n(t)$ and $\langle \alpha _n(t)\alpha _m(0)\rangle = 0$. In the purely harmonic approximation, one would have $g_n^ \circ (t) = \frac{{k_{\mathrm{B}}T}}{{\omega _n}}{\mathrm{e}}^{i\omega _nt}$. Anharmonic effects broaden the vibrational lines by a finite line-width*, γ*_*n*_, which results in a finite imaginary part of the frequency and in a decay of the single-mode Green’s function, reading: $g_n(t) = \frac{{k_{\mathrm{B}}T}}{{\omega _n}}{\mathrm{e}}^{i(\omega _n + i\gamma _n)t}$. By plugging this expressions into the lengthy formula that results from applying Wick’s theorem to the integrand of Eq. (1) and performing the time integral, after some cumbersome but straightforward algebra we get Eq. (3). A full derivation of Eqs. (3–5) is presented in the Supplementary Note 1.

To lowest order in the anharmonic interactions, vibrational linewidths can be computed from the classical limit of the Fermi golden rule, $\gamma _n$ = $\frac{{\pi \hbar ^2}}{{8\omega _n}}\mathop {\sum}\limits_{ml} {\frac{{|V{\prime\prime\prime}_{nml}|^2}}{{\omega _m\omega _l}}} \left[ {\frac{1}{2}(1 + n_m + n_l)\delta (\omega _n - \omega _m - \omega _l)} \right.$ + $\left. {(n_m - n_l)\delta (\omega _n + \omega _m - \omega _l)} \right]$, where *n*_*l*_ is the Bose-Einstein occupation number of the *l*-th normal mode and $V{\prime\prime\prime}_{nlm} = \frac{{\partial ^3V}}{{\partial \xi ^n\partial \xi ^l\partial \xi ^m}}$ is the third derivative of the potential energy with respect to the amplitude of the lattice distortion along the lattice normal modes^11^.

In order to show that our QHGK expression for the thermal conductivity, Eq. (3), reduces to the predictions of the BTE-RTA in crystals, we first notice that the *v*^*α*^ matrices of Eq. (4) can be expressed in terms of the matrix elements of the matrices $(V^\alpha )_{i\gamma }^{j\delta } = \frac{{R_{i\alpha }^ \circ - R_{j\alpha }^ \circ }}{{2\sqrt {M_iM_j} }}\Phi _{i\gamma }^{j\delta }$ between normal-mode eigenvectors: $v_{nm}^\alpha = (e_n,V^\alpha \cdot e_m)/\sqrt {\omega _n\omega _m}$, where the notations “(*e*, *e*′)” and “*V* ⋅ *e*” indicate scalar and matrix-vector products in the space of atomic displacements. In crystals, equilibrium atomic positions are characterized by a discrete lattice position, **a**_*i*_, and by an integer label, *s*_*i*_, indicating different atomic sites within a unit cell, **d**_*s*_: ${\mathbf{R}}_i^ \circ = {\mathbf{a}}_i + {\mathbf{d}}_{s_i}$. Likewise, in the Bloch representation, normal modes can be labeled by a quasi-discrete wavevector, **q**, belonging to the first Brillouin zone (BZ), and by a band index, *ν*: *n* → (**q**_*n*_, *ν*_*n*_). In particular, the IFC matrix and its eigenvectors can be expressed as $\frac{1}{{\sqrt {M_iM_j} }}\Phi _{j\beta }^{i\alpha } = \mathop {\sum}\limits_{\mathbf{q}} {{\mathrm{e}}^{i{\mathbf{q}}\cdot ({\mathbf{R}}_i^ \circ - {\mathbf{R}}_j^ \circ )}} D_{t\beta }^{s\alpha }({\mathbf{q}})$, where $D_{t\beta }^{s\alpha }({\mathbf{q}})$ is the dynamical matrix of the system and $\eta _{\nu {\mathbf{q}}}^{s\alpha }$ its eigenvectors: $e_{\nu}^\alpha = {\mathrm{e}}^{i{\mathbf{q}}_n\cdot {\mathbf{R}}_i^ \circ }\eta _{\nu _n{\mathbf{q}}_n}^{s_i\alpha }$ and $\mathop {\sum}\limits_{t\beta } {D_{t\beta }^{s\alpha }} ({\mathbf{q}})\eta _{\nu {\mathbf{q}}}^{t\beta } = \omega _{\nu {\mathbf{q}}}^2\eta _{\nu {\mathbf{q}}}^{s\alpha }$. When normal-mode eigenvectors are chosen to be real, the *v*^*α*^ matrices of Eq. (4) are real and anti-symmetric. In particular, $v_{nn}^\alpha = 0$ and a non-vanishing thermal conductivity results from the matrix elements of *v*^*α*^ connecting (quasi-) degenerate normal modes, i.e. modes whose frequencies coincide within the sum of their line widths. In the Bloch representation, *v*^*α*^ is anti-Hermitian and block-diagonal with respect to the wave-vector, **q**. Its diagonal elements are imaginary, though not necessarily vanishing. In this representation one has: $v_{\nu {\mathbf{q}},\mu {\mathbf{p}}}^\alpha = i\frac{{\delta _{{\mathbf{qp}}}}}{{\sqrt {\omega _{\nu {\mathbf{q}}}\omega _{\mu {\mathbf{p}}}} }}(\eta _{\nu {\mathbf{q}}},D^\alpha ({\mathbf{q}})\cdot \eta _{\mu {\mathbf{q}}})$, where $D^\alpha ({\mathbf{q}}) = \frac{{\partial D({\mathbf{q}})}}{{\partial q^\alpha }}$. At fixed **q**, the vibrational spectrum is strictly discrete i.e. it remains so even in the thermodynamic limit. The number of **q** points for which there exists a pair of distinct modes, (**q**, *ν*) and (**q**, *μ*) with *ν* ≠ *μ*, that are degenerate within the sum of their line-widths $(|\omega _{{\mathbf{q}}\nu } - \omega _{{\mathbf{q}}\mu }| \, \lesssim \, \gamma _{{\mathbf{q}}\nu } + \gamma _{{\mathbf{q}}\mu })$ is vanishingly small, because, in practice, this quasi-degeneracy can only occur close to high-symmetry lines. Furthemore, for such few pairs, one can prove that *v*_*ν***q**,*μ***q**_ ∝ *ω*_*ν***q**_ − *ω*_*μ***q**_. Hence in the periodic case the *τ*° matrix in Eq. (5) is strictly diagonal, $\tau _{{\mathbf{q}}\nu ,{\mathbf{p}}\mu }^ \circ = \delta _{{\mathbf{qp}}}\delta _{\nu \mu }\tau _{{\mathbf{q}}\nu }^ \circ$, where $\tau _{{\mathbf{q}}\nu }^ \circ = \frac{1}{{2\gamma _{{\mathbf{q}}\nu }}}$ is the anharmonic lifetime of the (**q**, *ν*) normal mode. We conclude that, for periodic systems in the Bloch representation, the double sum in Eq. (3) can be cast into a single sum over diagonal terms, reading: $\kappa _{\alpha \beta } = \mathop {\sum}\limits_{{\mathbf{q}}\nu } {v_{\nu {\mathbf{q}}}^\alpha } v_{\nu {\mathbf{q}}}^\beta \tau _{\nu {\mathbf{q}}}$, where $v_{\nu {\mathbf{q}}}^\alpha = \frac{1}{{2\omega _{\nu {\mathbf{q}}}}}(\eta _{\nu {\mathbf{q}}},D^\alpha ({\mathbf{q}})\cdot \eta _{\nu {\mathbf{q}}}) = \frac{{\partial \omega _{\nu {\mathbf{q}}}}}{{\partial q^\alpha }}$ is the group velocity of the *ν*-th phonon branch. This is the final expression for the thermal conductivity of a crystal in QHGK, which remarkably coincides with the solution of BTE-RTA^1^. We tested the QHGK approach against BTE-RTA for a crystalline silicon supercell of 1728 atoms, with a lattice parameter of 5.431 Å. The two calculations, performed with different codes, give the same results, as expected by the proven equivalence of the two methods for crystalline systems (see Supplementary Fig. 1).

Moving to the quantum regime is straightforward in our approach. To this end, we start from the quantum GK formula^3,5–8^, reading:

7 $$\kappa _{\alpha \beta } = \frac{1}{{VT}}\mathop {\int}\limits_0^{1/k_BT} {\mathrm{d}} \lambda \mathop {\int}\limits_0^{ + \infty } {\mathrm{d}} t\langle \hat J_\alpha (t + i\hbar \lambda )\hat J_\beta (0)\rangle ,$$

where $\hat J_\alpha$ are quantum heat-flux operators and 〈⋅〉 indicates quantum canonical averages. A quantum expression for the heat flux is obtained from its classical counterpart, Eq. (6), by making the substitutions: $\alpha \to \sqrt \hbar \hat a$ and $\alpha ^ \ast \to \sqrt \hbar \hat a^\dagger$, $\hat a^\dagger$ and $\hat a$ being normal-mode creation/annihilation operators, satisfying the standard commutation relations for bosons: $\left[ {\hat a,\hat a^\dagger } \right] = 1$. Note that no ordering ambiguities arise when quantizing Eq. (6) because the $v_{nm}^\alpha$ matrices are antisymmetric, and they therefore vanish for *n* = *m*. The resulting expression for the quantum heat flux is:

8 $$\hat J_\beta = \frac{{i\hbar }}{2}\mathop {\sum}\limits_{nm} {v_{nm}^\beta } \omega _m(\hat a_n^\dagger + \hat a_n)(\hat a_m^\dagger - \hat a_m).$$

The computation of the heat conductivity proceeds exactly as in the classical case, except for the expressions for the single-mode Green’s functions. In the quantum case they read: $\langle \hat a_n^\dagger (t)\hat a_n(0)\rangle = n_n{\mathrm{e}}^{i(\omega _n + i\gamma _n)t}$ and $\langle \hat a_n(t)\hat a_n^\dagger (0)\rangle = (n_n + 1){\mathrm{e}}^{ - i(\omega _n - i\gamma _n)t}$, $n_n = 1/\left( {e^{\frac{{\hbar \omega _n}}{{k_BT}}} - 1} \right)$ being the Bose-Einstein distribution function. The final quantum-mechanical expression for the heat conductivity in the QHGK is:

9 $$\kappa _{\alpha \beta } = \frac{1}{V}\mathop {\sum}\limits_{nm} {c_{nm}} v_{nm}^\alpha v_{nm}^\beta \tau _{nm}^ \circ ,$$

with $c_{nm} = \frac{{\hbar \omega _n\omega _m}}{T}\frac{{n_n - n_m}}{{\omega _m - \omega _n}}$. For *n* = *m* this term reduces to the modal heat capacity $c_n = k_{\mathrm{B}}\left( {\frac{{\hbar \omega _n}}{{k_{\mathrm{B}}T}}} \right)^2\frac{{e^{\hbar \omega _n/k_{\mathrm{B}}T}}}{{({\mathrm{e}}^{\hbar \omega _n/k_{\mathrm{B}}T} - 1)^2}}$. The other symbols are the same as in Eqs. (4) and (5) for the classical case. $\tau _{nm}^ \circ$, in particular, is only different from zero for $|\omega _n - \omega _m| \, \lesssim \, \gamma _n + \gamma _m$. Following the same derivation as for the classical case, one can prove that for periodic crystals Eq. (9) reduces to BTE-RTA. Furthermore, in the classical limit, one has lim_*T*→∞_ *c*_*nn*_ = *k*_B_ and the quantum formula, Eq. (9), reduces to Eq. (3). Further details are given in Supplementary Note 2.

### Simulations

**Fig. 1:**
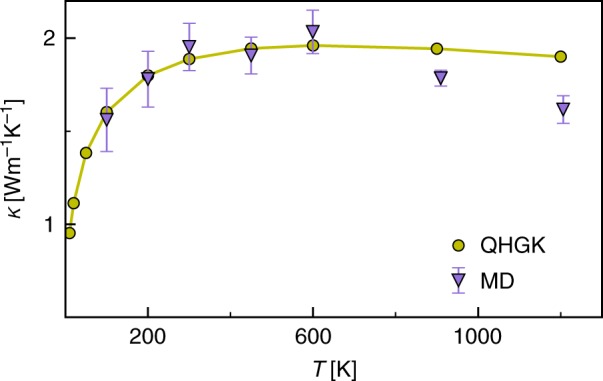
Classical thermal conductivity. Comparison between the thermal conductivity of a-Si computed for a 1728-atom supercell by the classical Green–Kubo theory of linear response using either our QHGK approach (Eq. (3), green) or equilibrium molecular dynamics (purple). The vertical bars indicate statistical errors obtained by means of cepstral analysis, as explained in Ref. ^22^. The *k*_*xx*_, *k*_*yy*_ and *k*_*zz*_ components of thermal conductivity tensor *κ* are averaged to obtain a value corresponding to an isotropic amorphous media

We validate our QHGK approach by testing the results of Eqs. (3) and (9) against MD simulations for amorphous silicon. Interatomic interactions are modeled using the empirical bond-order Tersoff potential^16^, which describes well the thermal conductivity of bulk and nanostructured silicon, including a-Si^9,10,17–19^. We consider a 1728-atom model of a-Si, generated by MD by quenching from the melt. Several EMD simulations where then run at different temperatures, as described in SM^20,21^. The integral of the heat flux autocorrelation function in Eq. (1) can then be efficiently evaluated via cepstral analysis, as described in refs. ^3,22^, which can be enhanced by averaging over multiple trajectories at low temperature (*T* ≤ 300 K). Details on the data analysis procedure followed here and on the estimate of the statistical errors is given in the Supplementary Note 3. The results of these calculations are reported in Fig. 1 and exhibit a weak non-monotonic dependence on *T*. Performing similar MD simulations on models of increasing size (4096 and 13,824 atoms) generated with the same protocol, we have verified that size effects on *κ* at 300 K amount to less than 15%, which is of the same order as the variation *κ* among different models with the same size. The computation of the IFC matrix, normal-mode frequencies and lifetimes is described in detail in SM, where we also display the resulting dependence of lifetimes on temperature (Supplementary Fig. 2). The resulting strongly diagonally dominant form of the *τ*° matrices in Eq. (5) is also displayed in Supplementary Fig. 2.

The thermal conductivity obtained by QHGK is in excellent agreement with that computed by EMD for *T* ≤ 600 K (Fig. 1). At higher temperatures QHGK overestimates *κ*, as it neglects higher-order anharmonic effects. We point out that, in spite of the formal analogies with the AF model^9,10,23^ and recent refinements thereof^24,25^, Eq. (3) entails no empirical parameters. It thus allows one to predict temperature trends dictated by anharmonic effects in good agreement with MD, without making any a priori distinction among propagating, diffusive, or localized vibrational modes. Conversely, in the AF model the temperature dependence lies only in the heat capacity term, therefore in the classical limit *κ* is temperature independent.

Similarly to the GK modal analysis approach^26^, based on classical MD, the transport character of the modes is dictated by the relative contribution from the diagonal and slightly off-diagonal terms of the $v_{nm}^\alpha$ matrix, weighted by $\tau _{nm}^ \circ$ (Supplementary Fig. 2). The generality of QHGK is expected to have a major impact for the study of weakly disordered systems, which are beyond the scope of applicability of approaches based on the BTE and the AF model.

**Fig. 2:**
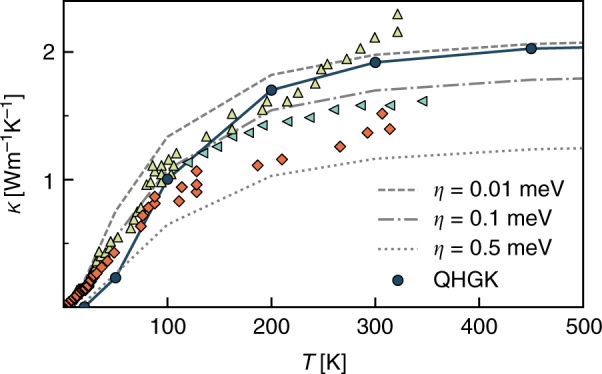
Quantum thermal conductivity. Thermal conductivity computed for a 13824-atom supercell of a-Si using the quantum QHGK approach in the quantum regime (Eq. (9)), compared with the Allen-Feldman approach^9,10,23^ and experimental data (yellow triangles and orange diamonds ref. ^27^), (green triangles ref. ^28^). The broadening *η* used in Allen-Feldman calculations is set equal for every normal mode

**Fig. 3:**
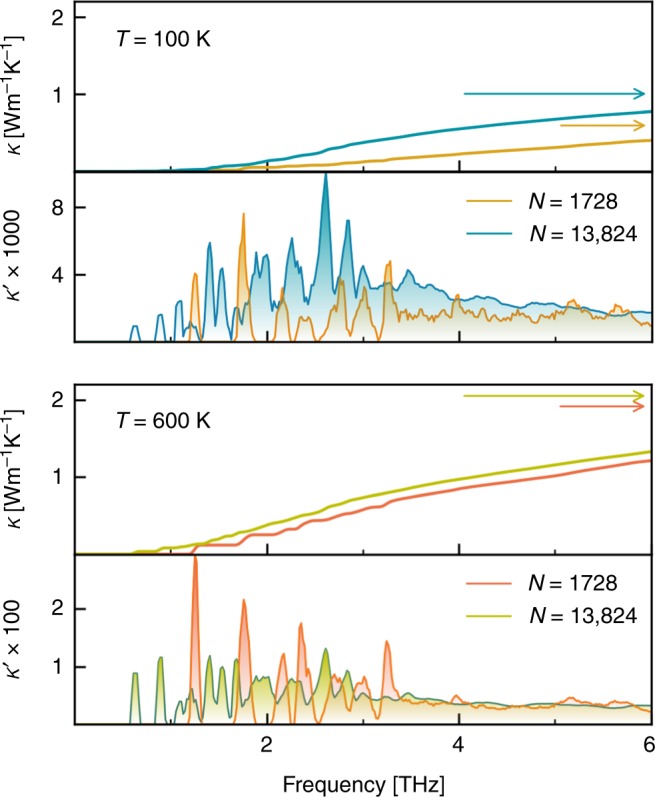
Thermal conductivity accumulation function. Conductivity accumulation function, *κ*(*ω*), and frequency-resolved differential conductivity, *κ*′(*ω*) (Eqs. (10) and (11)), computed for two different model sizes (*N* = 1,728 and *N* = 13,824 atoms) at temperatures *T* = 100 K and *T* = 600 K. Horizontal arrows in the upper panels indicate cumulative values of *κ*. *κ*′ is in units of WK^−1^ m^−1^ ps

QHGK is a general theory that allows one to accurately calculate thermal transport in both crystals and glasses at a full quantum mechanical level. In Fig. 2 we report our results from quantum QHGK calculation for an amorphous Si model of 13824 atoms along with three sets of experimental data^27,28^. QHGK results are in excellent agreement with the measurements in Ref. ^27^ above 100 K. At lower temperature the estimate of *κ* is affected by finite size effects, related to insufficient sampling of low-frequency acoustic modes: at 25 K these effects are so important, as to make the estimated conductivity almost vanish (see below). Specifically, we see a significant improvement in the estimate of *κ* at 50 K from the 1728-atom model (*κ* = 0.027 Wm^−1^ K^−1^) to the 13824 model (*κ* = 0.25 Wm^−1^ K^−1^ see Fig. 3). However, at 50 K and lower temperatures the latter is not converged yet. In fact, in order to eliminate finite-size effects, in our approach it would be necessary that in any relevant frequency range the density of vibrational states is larger than the normal-mode lifetimes, so that as many quasi-discrete normal modes as possible fall withing a line-width. In the low-frequency region, which is the most populated one in the quantum regime, this condition is hindered by the vanishing of both the density of states per unit volume and normal-mode line-widths. This effect is showcased in Fig. 3, where we compare for different temperatures and model sizes the frequency-resolved differential conductivity,

10 $$\kappa \prime (\omega ) = \frac{1}{{3V}}\mathop {\sum}\limits_\alpha {\mathop {\sum}\limits_{nm} \Delta } (\omega - \omega _n)c_{nm}(v_{nm}^\alpha )^2\tau _{nm}^ \circ ,$$

where Δ(*ω*) is a broadened approximation of the *δ* function and the other symbols have the same meaning as in Eq. (9), and the conductivity accumulation function defined as:

11 $$\kappa (\omega ) = {\int}_0^\omega {\kappa \prime } (\omega \prime ){\mathrm{d}}\omega \prime .$$

The AF model can also reproduce *κ* for a-Si, but it is extremely sensitive to the empirical choice of the line broadening parameter (*η*). The impact of *η* on the resulting *κ*(*T*) is also shown in Fig. 2, which shows that the value of *κ*_*AF*_ varies by a factor two by varying *η* between 0.01 and 0.5 meV in the temperature range considered. Whatever value is chosen for *η*, the AF model cannot reproduce the correct *κ*_*QM*_(*T*) of a-Si over the whole temperature range, in which we deem QHGK accurate (*T* ≤ 600 K), and it cannot give the correct decreasing trend at high temperature by construction. The predictions of the QHGK for the thermal conductivity of a-Si in the classical and fully quantum-mechanical regimes are compared in Supplementary Fig. 3.

### Conclusions

In conclusion, we have introduced a unified approach to compute the lattice thermal conductivity of both amorphous and crystalline systems. This quasi harmonic approach connects in a seamless fashion the AF model for disordered systems and the BTE-RTA for crystals. QHGK provides a significant improvement in generality over the Allen-Feldman model for disordered systems and is analytically proven to be equivalent to BTE for periodic systems. Classical QHGK calculations were validated against MD simulations for a-Si, and yield satisfactory agreement over a wide temperature range. Quantum QHGK can be deemed predictive at low temperature, not only for glasses and crystals but also for partially disordered systems, for which parameter-free models were up to now unavailable. Although the numerical results of this work were obtained by evaluating Eqs. (3)–(5) using equilibrium positions $R_{i\alpha }^ \circ$, second order force constants $\Phi _{i\beta }^{j\gamma }$, and line-widths *γ*_*n*_, computed at mechanical equilibrium (*T* = 0), it is possible to evaluate these same quantities through temperature-dependent statistical sampling approaches^29–31^, thus extending the reach of QHGK to systems with strong anharmonicity and high-temperature phases. The technique proposed in this work paves the way to robust calculations of heat transport in systems with any kind of structural order, including materials with point defects, extended defects and nanostructuring, without relying on any implicit knowledge of either their underlying symmetry, or the character of the vibrational modes, and without empirical parameters. While this paper was being written we learnt that conclusions similar to ours were reached by Simoncelli et al., following a different approach based on a generalization of the BTE^32^.

## Supplementary information

Supplementary Information

## Data Availability

The data that support the plots within this paper are available from the corresponding author upon reasonable request.

## Code availability

Computer codes are available from the corresponding author upon reasonable request.
