# Supplementary material for: Modeling heat transport in crystals and glasses from a unified lattice-dynamical approach
Source: Nat Commun. 2019 Aug 26;10:3853. doi: 10.1038/s41467-019-11572-4 (PMC6710279; doi:10.1038/s41467-019-11572-4)
Supplement: Supplementary file 1 — Supplementary Information [file 41467_2019_11572_MOESM1_ESM.pdf]

Modeling heat transport in crystals and glasses from a unified lattice-dynamical approach: Supplemental Material

Leyla Isaeva *et al.*

SUPPLEMENTARY NOTE 1 – THERMAL CONDUCTIVITY IN THE CLASSICAL QHGK

In order to establish Eqs. (3-5), we start from the expression for the harmonic heat flux, J_α, Eq. (6), and Hamiltonian, H, in terms of the normal-mode complex amplitudes defined in the text:

$$\xi_n = \sum_{i\alpha} \sqrt{M_i} u_{i\alpha} e^\alpha_{ni}; \qquad \pi_n = \sum_{i\alpha} \frac{1}{\sqrt{M_i}} \dot{u}_{i\alpha} e^\alpha_{ni}; \qquad \alpha_n = \sqrt{\frac{\omega_n}{2}} \xi_n + \frac{i}{\sqrt{2\omega_n}} \pi_n;$$
$$H = \frac{1}{2} \sum_n \left(\pi_n^2 + \omega_n^2 \xi_n^2 \right) = \sum_n \omega_n |\alpha_n|^2; \qquad J = \frac{i}{2} \sum_{nm} v_{nm} \omega_m (\alpha_n^* + \alpha_n)(\alpha_m^* - \alpha_m), \tag{1}$$

where the Cartesian index of the flux in the second line of Supplementary Equation (1) has been overlooked not to mess with the notation of the complex amplitudes. The time evolution of the α amplitudes is:

$$\alpha_n(t) = \alpha_n \mathrm{e}^{i\omega_n t}, \tag{2}$$

where $\alpha_n = \alpha_n(0)$ is the initial condition. The product of the two fluxes appearing in Eq. (1) is a fourth-order polynomial in the α's and α^*'s with time-dependent coefficients:

$$J(t)J(0) = P_4(\alpha, \alpha^*; t) = -\frac{1}{4} \sum_{mnpq} v_{nm} \omega_m v_{pq} \omega_q (\alpha_n^*(t) + \alpha_n(t))(\alpha_m^*(t) - \alpha_m(t))(\alpha_p^* + \alpha_p)(\alpha_q^* - \alpha_q). \tag{3}$$

The canonical average of the above polynomial with respect to the harmonic Hamiltonian in Supplementary Equations (1) is a Gaussian integral that can be evaluated using the Wick's theorem [1], stating that the canonical average of a fourth-order monomial is equal to the sum of all the possible contractions:

$$\langle ABCD \rangle = \langle AB \rangle \langle CD \rangle + \langle AC \rangle \langle BD \rangle + \langle AD \rangle \langle BC \rangle, \tag{4}$$

where any of the capital letters above indicate any complex amplitude, α_n or α_n^*. The relevant contractions are:

$$\langle \alpha_n \alpha_m \rangle = 0; \qquad \langle \alpha_n^*(t) \alpha_m \rangle = \delta_{mn} g_n(t), \tag{5}$$

where we define a single-mode classical Green's function $g_n(t) = \frac{k_B T}{\omega_n} e^{i\omega_n t}$. Hence, out of 16 terms in Supplementary Equation (3) only 6 are non vanishing. One such non-vanishing term is $\langle \alpha_n(t) \alpha_m(t) \alpha_p^* \alpha_q^* \rangle$, and the others are obtained by keeping two of the complex amplitudes conjugated. Making use of Wick's theorem this fourth-order correlator is reduced to the sum of the two terms:

$$\langle \alpha_n(t) \alpha_m(t) \alpha_p^* \alpha_q^* \rangle = \langle \alpha_n(t) \alpha_p^* \rangle \langle \alpha_m(t) \alpha_q^* \rangle + \langle \alpha_n(t) \alpha_q^* \rangle \langle \alpha_m(t) \alpha_p^* \rangle = (\delta_{np}\delta_{mq} + \delta_{nq}\delta_{mp}) g_n(t) g_m(t). \tag{6}$$

Working out the rest of the canonical averages in Supplementary Equation (3), we may obtain the following relation for the left-hand side of the Supplementary Equation (3) in terms of single-mode classical Green's functions $g_n(t)$ and $g_m(t)$:

$$\langle J(t)J(0) \rangle = -\frac{1}{4} \sum_{nm} \left(\frac{\omega_n - \omega_m}{\omega_n} (g_n(t) g_m(t) + g_n^*(t) g_m^*(t)) - \frac{\omega_n + \omega_m}{\omega_n} (g_n(t) g_m^*(t) + g_n^*(t) g_m(t)) \right). \tag{7}$$

Introducing anharmonicity into our quasi-harmonic treatment through the linewidths, γ_n, of the vibrational normal modes results in the decay of the single-mode Green's functions $g_n(t)$ as:

$$g_n(t) = \frac{k_B T}{\omega_n} e^{i(\omega_n + i\gamma_n)t}. \tag{8}$$

By performing the time integrations and symmetrizing the final results, one obtains the heat conductivity tensor, represented in term of matrices v_{nm} as

$$\kappa_{\alpha\beta} = \frac{k_B}{V} \sum_{nm} v^\alpha_{nm} v^\beta_{nm} \tau_{nm}, \qquad v^\alpha_{nm} = \frac{1}{2\sqrt{\omega_n \omega_m}} \sum_{ij\beta\gamma} \frac{R^\circ_{i\alpha} - R^\circ_{j\alpha}}{\sqrt{M_i M_j}} \Phi^{j\gamma}_{i\beta} e^{i\beta}_n e^{j\gamma}_m, \tag{9}$$

and matrix τ_{nm} given by the sum of two Lorentzian functions:

$$\tau_{nm} = \frac{(\omega_n + \omega_m)^2}{4\omega_n \omega_m} \frac{\gamma_n + \gamma_m}{(\gamma_n + \gamma_m)^2 + (\omega_n - \omega_m)^2} + \frac{(\omega_n - \omega_m)^2}{4\omega_n \omega_m} \frac{\gamma_n + \gamma_m}{(\gamma_n + \gamma_m)^2 + (\omega_n + \omega_m)^2}. \tag{10}$$

In the quasi-harmonic regime, linewidths are much smaller than normal-mode frequencies: $\epsilon = \frac{\gamma}{\omega} \ll 1$. In this regime, the second "*antiresonant*" term in Supplementary Equations (10) can be neglected with respect to the first. To the same order in ϵ, one has: $\frac{(\omega_n+\omega_m)^2}{4\omega_n\omega_m} \approx 1 + \left(\frac{\omega_n-\omega_m}{\omega_n+\omega_m}\right)^2$. By substituting this expression into Supplementary Equations (10), one gets: $\tau_{nm} = \tau^\circ_{nm} + \mathcal{O}\left(\epsilon^2\right)$, *cfr.* Eq. (5).

SUPPLEMENTARY NOTE 2 – THERMAL CONDUCTIVITY IN THE QUANTUM QHGK

The derivation of the quantum QHGK expression for the heat conductivity follows the same path as in the classical case. To complete this derivation, we first introduce the quantum propagators $G_n(t)$ and $\tilde{G}_n(t)$ by promoting the classical complex amplitudes α_n, α^*_m to the quantum ladder operators $\alpha_n \to \sqrt{\hbar}a_n, \alpha^*_m \to \sqrt{\hbar}a^\dagger_m$ satisfying the Bose-Einstein commutation rule $[a_n, a^\dagger_m] = \delta_{nm}$:

$$G_n(t) = \hbar\langle a^\dagger_n(t)a_n(0)\rangle = \hbar n_k e^{i\omega_n t}, \qquad \tilde{G}_n(t) = \hbar\langle a_n(t)a^\dagger_n(0)\rangle = \hbar(n_k+1)e^{-i\omega_n t}. \tag{11}$$

We note that in the high-temperature limit the quantum single-mode Green's functions reduce to the classical one $\lim_{\hbar\to 0} G_n(t) = \lim_{\hbar\to 0} \tilde{G}^*_n(t) = g_n(t)$. Next, in analogy with the classical case, we write the quantum canonical average $\langle\hat{J}(t)\hat{J}(0)\rangle$:

$$\langle\hat{J}(\tau)\hat{J}(0)\rangle = -\frac{1}{4}\sum_{nm}\left(\frac{\omega_n-\omega_m}{\omega_n}\left(G_n(\tau)G_m(\tau)+\tilde{G}_n(\tau)\tilde{G}_m(\tau)\right) - \frac{\omega_n+\omega_m}{\omega_n}\left(G_n(\tau)\tilde{G}_m(\tau)+\tilde{G}_n(\tau)G_m(\tau)\right)\right), \tag{12}$$

where $\tau = t + i\hbar\lambda$ is the complex argument of the quantum GK formula, Eq. (7). By introducing finite mode linewidths and performing the double time integration in Eq. (7), we arrive at the following lengthy relation for the thermal conductivity tensor:

$$\kappa_{\alpha\beta} = \frac{\hbar^2}{4VT}\sum_{nm} v^\alpha_{nm} v^\beta_{nm}\left(\frac{n_n-n_m}{\hbar(\omega_n-\omega_m)}\frac{\gamma_n+\gamma_m}{(\omega_n-\omega_m)^2+(\gamma_n+\gamma_m)^2}(\omega_n+\omega_n)^2\right.$$
$$\left. + \frac{e^{\beta\hbar(\omega_n+\omega_m)}-1}{\hbar(\omega_n+\omega_m)}\frac{2(\omega_n-\omega_m)^2}{(e^{\beta\hbar\omega_n}-1)(e^{\beta\hbar\omega_m}-1)}\frac{\gamma_n+\gamma_m}{(\omega_n+\omega_m)^2+(\gamma_n+\gamma_m)^2}\right). \tag{13}$$

The second, antiresonant, term in Supplementary Equation (13) can be neglected in the quasi-harmonic regime $\left(\frac{\gamma}{\omega} \to 0\right)$, while the first one can be cast into a BTE-like form by introducing the matrix $c_{nm} = \frac{\hbar\omega_n\omega_m}{T}\frac{n_n-n_m}{\omega_n-\omega_m}$:

$$\kappa_{\alpha\beta} = \frac{1}{V}\sum_{nm} c_{nm} v^\alpha_{nm} v^\beta_{nm} \tau^\circ_{nm}. \tag{14}$$

SUPPLEMENTARY NOTE 3 – COMPUTATIONAL DETAILS

A liquid model prepared at 3000 K is quenched and then equilibrated to 2000 K for 10 ns at constant pressure. It is then further quenched to 300 K at constant volume in 10 ns, and equilibrated at the same temperature for 1 ns. This procedure produces a-Si models of good quality with a very low concentration of coordination defects [2]. The model is a cubic simulation box with a density of 2.3 g/cm^3. The resulting radial and bond-angle distribution functions are reported in Supplementary Figure 4. The average coordination is 4.06 neighbours per atom, indicating that the system can be considered as a random tetrahedral network.

For this model we calculate κ at several temperatures between 100 K and 1200 K by equilibrium MD simulations implementing Eq.(1) according to GK theory. Starting from the model at 300 K, the system is equilibrated at the target temperatures for 1 ns at fixed volume before each production run. The latter is carried out integrating the equations of motion in the microcanonical ensemble (NVE) with a timestep of 0.5 fs for a total of 25 ns. All MD simulations are performed using the GPUMD open-source code [3], calculating the heat flux J every 4 fs [4].

The thermal conductivity was extracted from the energy flux thus generated, using the recently introduced *cepstral analysis* method [5, 6]. Cepstral analysis [7] is a technique, commonly used in signal analysis and speech recognition, to process the power spectrum of a time series, leveraging its smoothness and the statistical properties of its samples. According to Eq. (1) of the main text, the thermal conductivity is proportional to the zero-frequency value of the power

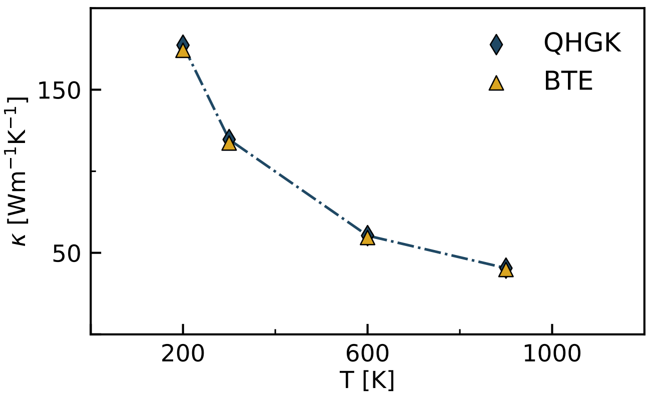

Supplementary Figure 1. **Comparison between QHGK approach and conventional BTE calculations.** Thermal conductivity of fcc Si computed for a 1728-atom supercell using our QHGK approach (blue) in comparison with the standard BTE calculations (yellow).

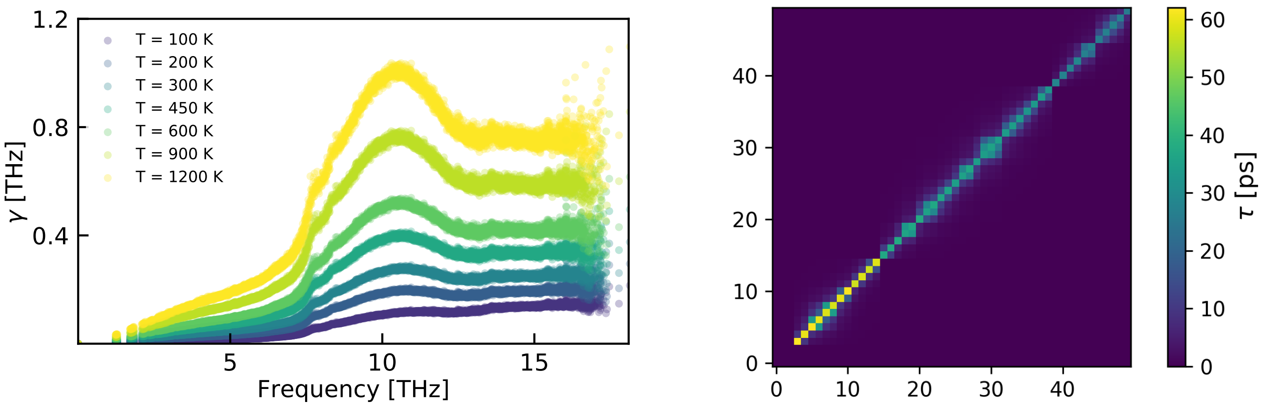

Supplementary Figure 2. **Numerical calculations of normal-mode linewidths.** Left: Normal-mode linewidths of 1728-atomic model of a-Si computed for various temperatures using Fermi golden rule and the classical limit of the Bose-Einstein occupation function, which corresponds to equipartition. Right: Partial heatmap of matrix τ_{nm} computed for T=300 K. The structure of the matrix, *i.e.* non-vanishing diagonal and close-to-diagonal elements, is dictated by its analytical form given by the Lorentzian function.

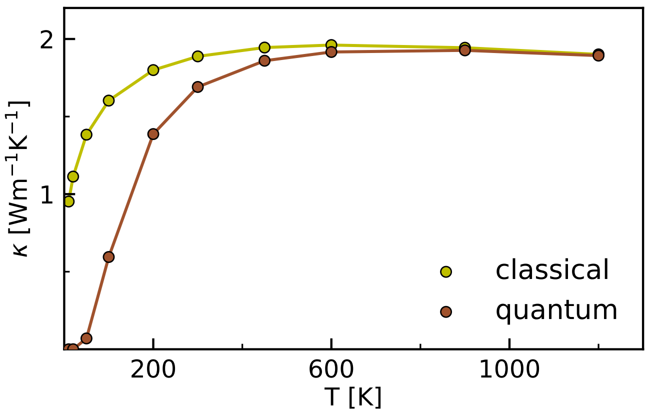

Supplementary Figure 3. **Comparison between the predictions of the QHGK in classical and quantum regimes.** Thermal conductivity computed for 1728-atomic model of a-Si is shown for both classical and fully quantum-mechanical regimes.

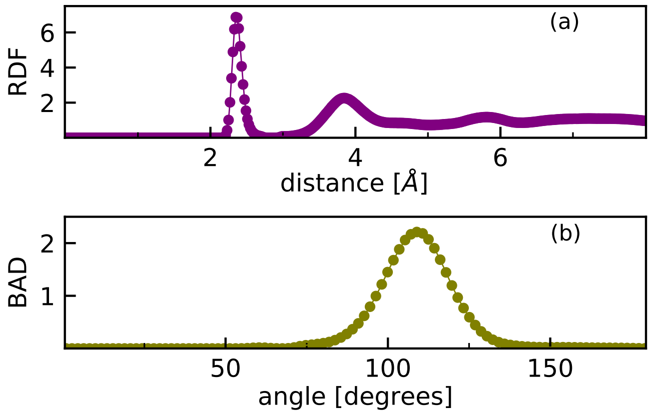

Supplementary Figure 4. **Structural properties of a-Si model.** (a) Radial distribution function (RDF) and (b) bond angle distribution (BAD) of the 1728 atoms model of a-Si used for the calculations of thermal conductivity.

spectrum of the energy flux: $\kappa \propto S(\omega = 0)$, where $S(\omega) = \int_{-\infty}^{\infty} \mathrm{e}^{i\omega t} C(t) dt$, and $C(t) = \langle J(t) J(0) \rangle$ is the flux time auto-correlation function. The Wiener-Kintchnine theorem [8] states that $S(\omega)$ is asymptotically proportional to the expectation of the squared modulus of the truncated Fourier transform of the flux sample: $S(\omega) = \lim_{\tau\to\infty} \langle \frac{1}{\tau} |\tilde{J}_\tau(\omega)|^2 \rangle$, where $\tilde{J}_\tau(\omega) = \int_0^\tau J(t) \mathrm{e}^{i\omega t} dt$. In the long-time limit, the squared modulus to be averaged is a stochastic process whose values are independent for $\omega \neq \omega'$ and individually distributed as $\frac{1}{\tau}|\tilde{J}_\tau(\omega)|^2 = S(\omega)\xi(\omega)$, where $\xi(\omega) \sim \frac{1}{2}\chi_2^2$, χ_2^2 being a chi-square variate with two degrees of freedom. The multiplicative nature of the noise affecting the sample spectrum suggests that the power of the noise can be reduced by applying a low-pass filter to its logarithm. This is the main idea underlying cepstral analysis, which can be leveraged to devise a consistent and asymptotically unbiased estimator for the the zero-frequency value of the flux power spectrum, which is proportional to the transport coefficient we are after. For more details, see Refs. 5, 9, and 6. Given the strongly harmonic nature of the system at low and intermediate temperatures, in order to improve the sampling of the phase space at 300 K and below, we average the results obtained by cepstral analysis over two independent simulations 25 ns long.

In order to implement the classical QHGK approach as in Eq. (4), we optimize the a-Si model structure by steepest descent and calculate the second- and third-order force constant matrices by finite differences (frozen phonon method) with atoms displacements of 10^{-4} Å. Normal modes line widths γ_n, necessary to evaluate Eq. (5) for τ_{nm}, are computed using the Fermi golden rule [10] (See Supplementary Figure 2). In the disordered case, this is done explicitly only for the smaller (1728-atom) sample. For larger samples, we interpolate the inverse linewidth, i.e. lifetimes, as a function of frequency from the explicit results for the 1728 atoms system. Lifetimes vs. frequencies are averaged over frequency bins and then interpolated with third-order splines, with the constraint that at low frequency $\tau \propto 1/\omega^2$ [11]. When comparing with classical MD simulations, QHGK results were obtained using the classical limit of the normal-mode lifetimes; the full quantum expression was used otherwise.

SUPPLEMENTARY REFERENCES

[1] Negele, J. W. & Orland, H. *Quantum Many-particle Systems* (Perseus Books, 1988).
[2] Deringer, V. L. *et al.* Realistic Atomistic Structure of Amorphous Silicon from Machine-Learning-Driven Molecular Dynamics. *J. Phys. Chem. Lett.* **9**, 2879–2885 (2018).
[3] Fan, Z., Chen, W., Vierimaa, V. & Harju, A. Efficient molecular dynamics simulations with many-body potentials on graphics processing units. *Comp. Phys. Commun.* **218**, 10 (2017). URL https://www.sciencedirect.com/science/article/pii/S0010465517301339.
[4] Fan, Z. *et al.* Force and heat current formulas for many-body potentials in molecular dynamics simulations with applications to thermal conductivity calculations. *Phys. Rev. B* **92**, 094301 (2015). URL https://doi.org/10.1103/PhysRevB.92.094301.
[5] Ercole, L., Marcolongo, A. & Baroni, S. Accurate thermal conductivities from optically short molecular dynamics simulations. *Sci. Rep.* **7**, 15835 (2017). URL https://www.nature.com/articles/s41598-017-15843-2.
[6] Bertossa, R., Grasselli, F., Ercole, L. & Baroni, S. Theory and numerical simulation of heat transport in multi-component systems (2018). arXiv:1808.03341.
[7] Bogert, B. P., Healy, J. R. & Tukey, J. W. The quefrency analysis of time series for echoes: Cepstrum, pseudo-

autocovariance, cross-cepstrum, and saphe cracking. In *Proceedings of the Symposium on Time Series Analysis*, 209–243 (1963).
Childers, D. G., Skinner, D. P. & Kemerait, R. C. The cepstrum: A guide to processing. *Proceedings of the IEEE* **65**, 1428–1443 (1977).

[8] Wiener, N. Generalized harmonic analysis. *Acta Math.* **55**, 117–258 (1930).
Khintchine, A. Korrelationstheorie der stationären stochastischen Prozesse. *Math. Ann.* **109**, 604–615 (1934). URL `http://dx.doi.org/10.1007/BF01449156`.

[9] Baroni, S., Bertossa, R., Ercole, L., Grasselli, F. & Marcolongo, A. *Heat Transport in Insulators from Ab Initio Green-Kubo Theory*, 1–36 (Springer International Publishing, Cham, 2018), 2 edn. URL `https://doi.org/10.1007/978-3-319-50257-1_12-1`. 1802.08006.

[10] Fabian, J. & Allen, P. B. Anharmonic decay of vibrational states in amorphous silicon. *Phys. Rev. Lett.* **77**, 3839 (1996). URL `https://journals.aps.org/prl/abstract/10.1103/PhysRevLett.77.3839`.

[11] Asen-Palmer, M. *et al.* Thermal conductivity of germanium crystals with different isotopic compositions. *Phys. Rev. B* **56**, 9431–9447 (1997). URL `https://doi.org/10.1103/PhysRevB.56.9431`.
